# Isolation and characterisation of mouse intestinal mesoangioblasts

**DOI:** 10.1007/s00383-018-4373-7

**Published:** 2018-11-08

**Authors:** Silvia Perin, Conor J. McCann, Paolo De Coppi, Nikhil Thapar

**Affiliations:** 1 Stem Cells and Regenerative Medicine, UCL Great Ormond Street Institute of Child Health, 30 Guilford Street, London, WC1N 1EH UK; 2 Specialist Neonatal and Paediatric Surgery (SNAPS) Department, Great Ormond Street Hospital NHS Foundation Trust, London, UK; 3 Neurogastroenterology and Motility Unit, Department of Gastroenterology, Great Ormond Street Hospital NHS Foundation Trust, London, UK

**Keywords:** Mesoangioblasts, Smooth muscle cells, Tissue engineering, Small intestine, Regenerative medicine

## Abstract

**Aims and objectives:**

Children suffering from intestinal failure (IF) endure considerable morbidity and overall have poor survival rates, complicated by the shortage of organs available for transplantation. Therefore, new therapeutic approaches are pivotal if outcomes are to be improved. Over the past years, tissue engineering (TE) has emerged as a possible alternative treatment for many congenital and acquired conditions. TE aims at creating bioengineered organs by means of combining scaffolds with appropriate cell types, which in the intestine are organised within a multilayer structure. In order to generate functional intestine, this cellular diversity and organisation will need to be recreated. While the cells for the epithelial, neural and vascular compartments have been well defined, so far, less attention has been put on the muscular compartment. More recently, mesoangioblasts (MABs) have been identified as a novel source for tissue regeneration since they are able to give rise to vascular and other mesodermal derivatives. To date MABs have not been successfully isolated from intestinal tissue. Therefore, our aim was to demonstrate the possibility of isolating MABs from adult mouse small intestine.

**Materials and methods:**

All experiments were carried out using small intestinal tissues from C57BL/6J mice. We applied an established protocol for MAB isolation from the isolated neuromuscular layer of the small intestine. Cultured cells were stained for Ki67 to assess proliferation rates as well as for a panel of pericyte markers to determine their phenotype.

**Results:**

Cells were successfully isolated from gut biopsies. Cultured cells showed good proliferative capacity and positivity for at least three pericytes markers found in vessels of the gut neuromuscular wall: neuron-glial antigen 2, alkaline phosphatase and platelet-derived growth factor β.

**Conclusion:**

This proof-of-principle study lays the foundation for further characterization of MABs as a possible cell source for intestinal smooth muscle regeneration and TE.

## Introduction

The gastrointestinal tract (GI) is a complex physiological system composed of many organs. Its main function is to fulfil the nutritional demands of the body by processing food and eliminating waste, thanks to a muscular gut wall that mixes and moves luminal contents along the tubular organs [1]. When intestinal capacity to fulfil nutritional demands becomes insufficient and parenteral nutrition is needed, intestinal failure (IF) occurs [2]. IF afflicts ten of thousands of children worldwide [3] but with an overall mortality rate of around 25%, mainly due to multi-organ system failure, sepsis, haemorrhage caused by prolonged parenteral nutrition and complications associated with intestinal transplantation [4]. IF remains the primary indication for intestinal transplantation in children, particularly related to short bowel syndrome or gastrointestinal motility disorders [5]. Unfortunately, small bowel transplantation is still a very challenging approach: in children, isolated small bowel transplantation has shown very high incidence of acute rejection, up to 45% within the first two years, with a small reduction (around 35–38%) when multi-organ transplantation is performed [5]. Furthermore, the paucity of available, correctly sized organs for children, along with the necessity of life-long immunosuppression, poses additional obstacles. In this context, a tissue engineering (TE) approach could provide an alternative strategy to small bowel transplantation with the potential to overcome organ donor shortages and the necessity of life-long immunosuppression [6]. To generate a functional intestine with a TE approach, two critical steps need to be overcome: the generation of a biocompatible scaffold and the isolation, expansion (and subsequent seeding in the scaffold) of the different cell types that compose a functional intestine.

To date, researchers have attempted to regenerate several organs, including the intestine, using decellularised scaffolds [7–10]. Totonelli and colleagues have shown that decellularised intestine maintains critical intestinal extracellular components and reported epithelial cell adherence and preservation of angiogenic properties of decellularised scaffolds [9]. In regards to specific cellular components, the primary focus of recellularisation studies have been both the epithelial and vascular compartments as reported by Kitano et al., where intestinal organoids were used for the regeneration of a functional intestinal mucosa [8]. Less attention has been placed on the neuromuscular compartment with few studies reporting on the use of neural crest cells derived from induced pluripotent stem cells [11, 12]. Indeed, there have been no reported studies focussed on the muscular compartment in itself, highlighting the need for research into this under-represented, but critical, cell type for intestinal regeneration.

Recently, pericytes have been identified as a powerful cell source for tissue regeneration [13]. In fact, pericytes, specialised cells of the vessel mural wall defined by their position underneath the basal lamina of micro-vessels, have been indicated as multipotent stem/progenitor cells that resemble mesenchymal stem cells [14–17]. Amongst them, a group of cells able to give rise to vascular and other mesodermal derivatives in vivo [18] have been identified and termed mesoangioblasts (MABs). When derived from adult tissue, MABs lose endothelial properties but maintain features of pericytes, suggesting that they can be referred as pericyte-derived cells. The role of MABs as a tool for tissue regeneration has very recently been confirmed by Urbani et al., who showed that human MABs derived from skeletal muscle biopsies can contribute to the regeneration of the neuromuscular wall of bioengineered oesophagus [10]. It is therefore possible that the gut musculature may regenerate via a perycite-control mechanism. In this perspective, isolation of MABs from the intestine could improve the efficiency of muscular intestinal regeneration. The aim of this study was to test the possibility of isolating MABs from the musclular wall of the murine small intestine.

## Methods

### Mouse small intestine dissection

Small intestines were dissected from C57BL/6J mice, obtained from The Jackson Laboratory (Bar Harbor, MN, USA) after cervical dislocation. Animal house facilities were compliant with the UK Home Office Certificate of Designation and all animals used in the experiments were kept in conformity with the UK Animals Act 1986 and approved by the University College London Biological Services Ethical Review Process.

### Cells isolation

Cells were isolated with adaptations from the protocol published by Tonlorenzi et al. [19]. Following washes with sterile phosphate buffer saline (PBS, Gibco), smooth muscle layers of the small intestine were carefully dissected from the submucosal and mucosal layers, using fine forceps under a stereomicroscope. The intestine was opened and pinned out onto a Sylgard^®^ coated plate filled with PBS supplemented with 1% v/v Pen-Strep (Gibco) to prevent bacterial infection. Subsequently, the mucosa was delicately separated from the neuromuscular layer, which was then cut into 2 mm^2^ wide pieces. These pieces were transferred into 6-well dishes, pre-treated with 1% v/v growth factor reduced Matrigel™ (MRF, BD) to favour attachment and cell outgrowth, with a maximum of three pieces per well. Enriched Megacell medium (Sigma) was gently added, to avoid fragment detachment, until the muscle pieces were covered. Dishes were placed inside a humidified chamber and placed in an incubator at 37 °C, 5% CO_2_, 5% O_2_. The following day, fresh medium was added on each well.

Every 2 days, cultures were checked for preliminary growth and the medium was changed. After 6–10 days, according to the level of cell outgrowth, cells were trypsinized and placed into flasks. The medium composition for cell outgrowth and expansion was prepared by enriching Megacell with 5% v/v foetal bovine serum (FBS, Gibco), 1% v/v non-essential amino acids (Gibco), 1% v/v L-glutamine (Gibco), 1% v/v Pen-Strep (Gibco), 0.05 mM β-mercaptorthanol (Sigma) and 5 ng/ml basic-Fibroblast Growth Factor (Peprotec).

For cell expansion, when cells reached 60–70% confluency, they were detached, always recovering both the adherent and the floating population. After gentle rinsing with PBS, trypLE™ (Thermo Scientific) was added and incubated at 37 °C for 3 min, when all cells detached from the flask and split at approximately 1:5.

### Immunostaining

Cells and intestinal cryosections (10µm thick) were fixed for 8 min with 4% w/v paraformaldehyde solution (PFA, Sigma) and subsequently washed with PBS. Cell permeabilization and blocking were performed with 1% w/v Bovine Serum Albumin (BSA, Sigma)/0.1% v/v Triton-X in PBS for 1 h at room temperature. Samples were incubated overnight at 4 °C with primary antibodies (Table 1) diluted in 1% w/v BSA/ 0.1% v/v Triton-X in PBS, washed three times for 5 min with PBS and then detected with secondary antibodies (Table 1) diluted in 1% w/v BSA/ 0.1% v/v Triton-X in PBS for 1 h at room temperature. Images were taken using a Zeiss LSM710 confocal microscope and processed using the software ImageJ [20].

**Table 1:** List of antibodies used

Antigen	Host	Company	Cat. number	Concentration
Ki67	Rabbit	Abcam	ab15580	1:100
PDGFRβ	Rabbit	Abcam	ab32570	1:100
NG2	Rabbit	Millipore	ab5320	1:100
CD31	Mouse	Invitrogen	Ma5-13188	1:10
α Rb 488	Goat	Invitrogen	A-11034	1:500
α Ms 568	Goat	Invitrogen	A-11004	1:500
Hoechst 33342		ThermoFisher	H1399	1:500

### Alkaline phosphatase staining

Cells and intestinal cryosections were stained for alkaline phosphatase following Vector^®^ Red protocol (Vector^®^ Red Alkaline Phosphatase Substrate, Vector Laboratories). Briefly, a working solution was prepared by mixing Reagent 1, 2 and 3 with the buffer solution: 200 mM Tris–HCl (Sigma), pH 8.2–8.5. Samples were incubated with working solution for 30 min in the dark and then washed three times for 5 min with the buffer solution before being counter stained with Hoechst (ThermoFisher Scientific) for nuclear staining.

## Results

**Fig. 1:**
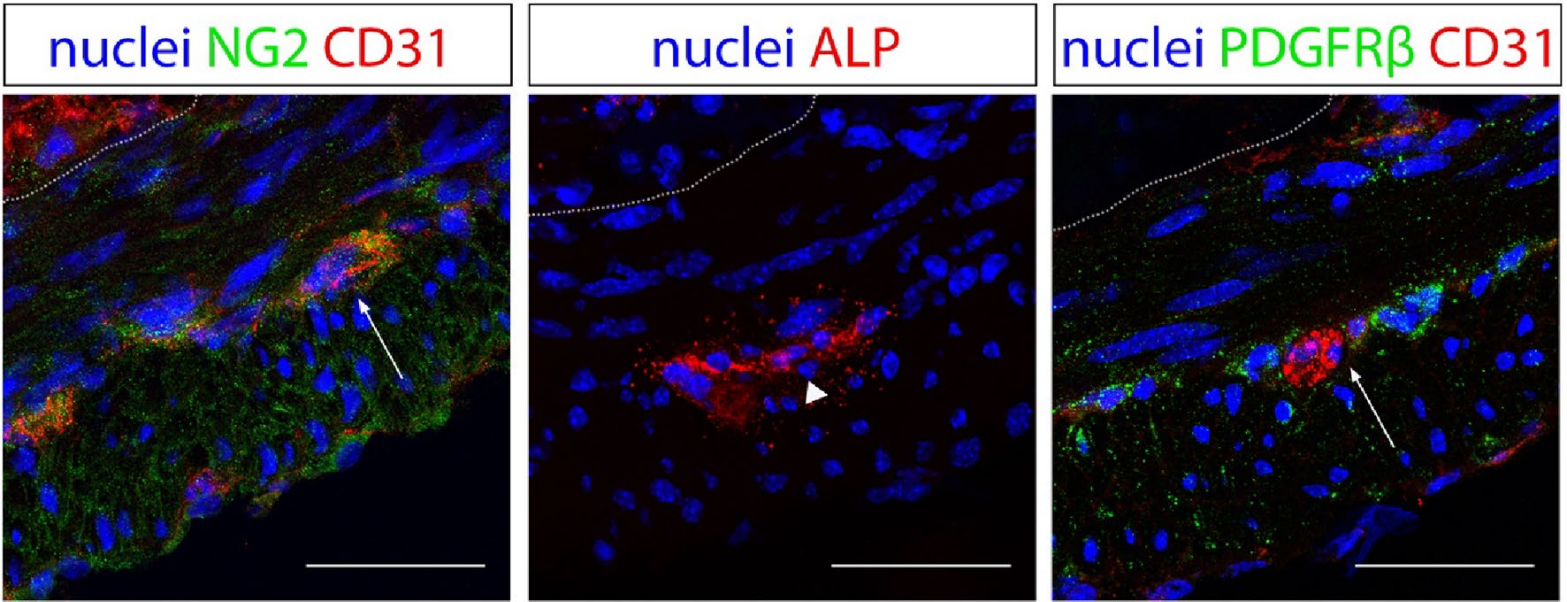
Immunoflourescence staining of mouse small intestinal sections. Muscle layers are located below the dotted lines. CD31 positive endothelial cells (red, right and left panels) are located between the two muscle layers and neuron-glial antigen 2 and platelet-derived growth factor β positive cells are localised in close proximity (arrows, green). Alkaline phosphatase positive cells in red are localised between the two muscle layers (arrowhead). Scale bar = 50 µm

In the neuromuscular gut wall, micro-vessels are mainly located between the circular and longitudinal muscle layer as shown by positivity for CD31, marker of endothelial cells (Fig. 1, arrows in right and left panels). As shown in Fig. 1, cells positive for neuron-glial antigen 2 and platelet derived-growth factor β were located in close proximity to CD31 positive cells (Fig. 1). Additionally, positivity for alkaline phosphatase was visible in the same region in between the two muscle layers of small intestinal wall (Fig. 1).

**Fig. 2:**
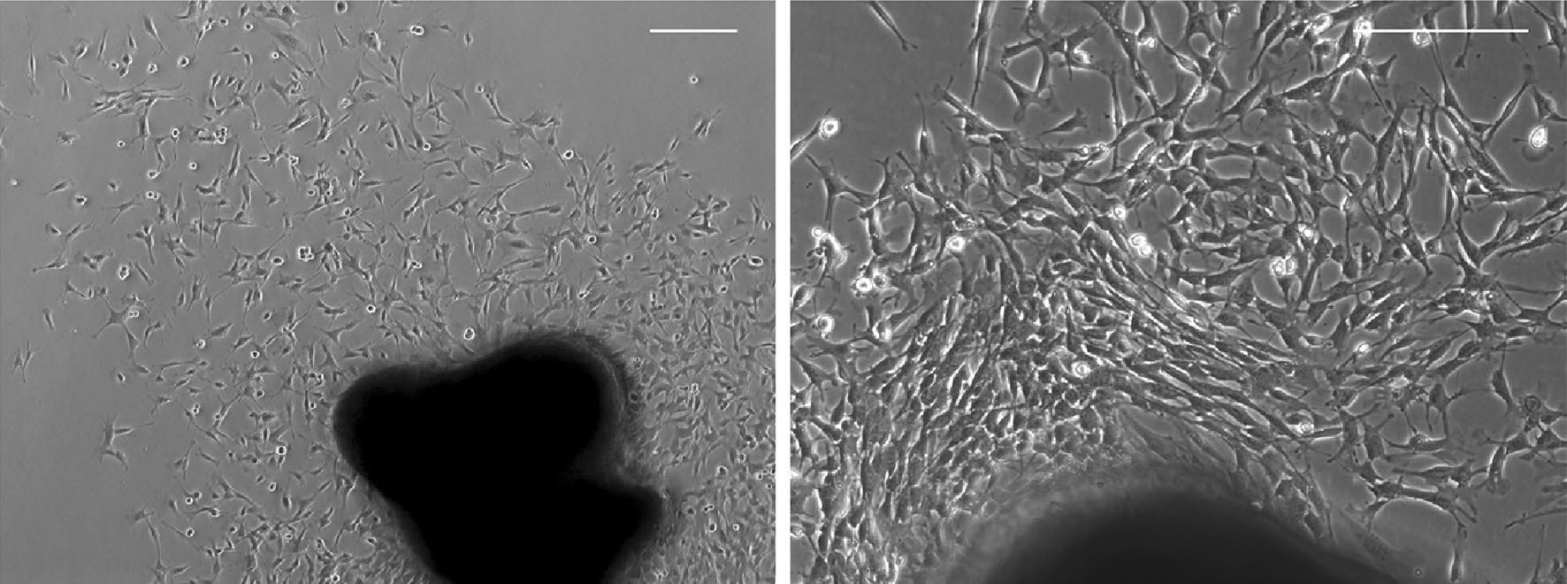
Cells spreading out from the biopsies (dark shapes at the bottom of the images) onto the plate. Scale bar = 200 µm

Cellular outgrowth from gut muscular biopsies was visible after 3–5 days of culture. Triangular shaped cells spread out from the intestinal muscle biopsy attached to the Matrigel™ reduced factor coated dish (Fig. 2). The cells were then passaged and cultured cells after the second passage had an average percentage of Ki67 positive cells of 28.6% ± 8% (*n* = 3 technical replicates).

**Fig. 3:**
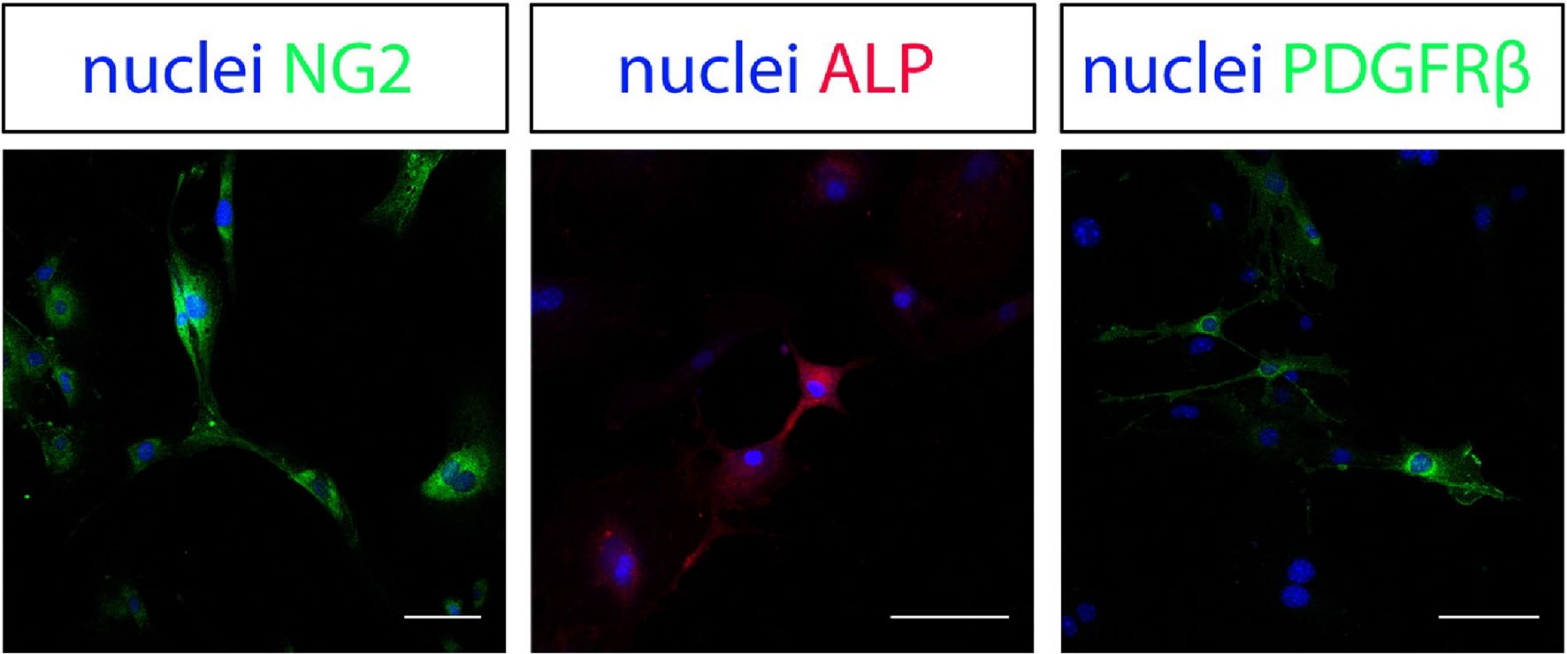
Cultured cells immunostained for neuron-glial antigen 2 (NG2) in green, alkaline phosphatase (ALP) in red and platelet-derived growth factor receptor β (PDGFR β) in green. Nuclei were stained with Hoechst in blue. Scale bar = 50 µm

Cultured cell were then characterised for the expression of a range of pericyte markers, which would confirm the origin of the isolated cells. Immunostaining of isolated cells after 2 passages in culture revealed positivity for neuron-glial antigen 2 (NG2), positivity for alkaline phosphatase (ALP) and platelet-derived growth factor β (PDGFRβ: Fig. 3).

## Discussion

Given the poor survival rate in children with IF together with a perpetual shortage of organs available for transplantation, alternative therapeutic approaches are vital for the treatment of this devastating condition if outcomes are to be improved. Recent advances in the fields of stem cell research and bioengineering have demonstrated the possibilities for tissue engineered organs in a number of systems [7, 8, 10, 21]. Indeed, development of a bio-engineered oesophagus for the possible treatment of oesophageal atresia and other acquired damages to the oesophagus may be possible thanks to MABs, which are capable of driving smooth muscle regeneration [10]. However, while tissue engineering may provide a mechanism of producing small-scale segments of recellularised tissue for transplantation, the development of large-scale functional segments of intestine for the treatment of IF is daunting. The intestine is a complex structure with absorptive and secretory functions that change along the length of the organ. Great progress has been made with regards to epithelial regeneration with the ability of derive organoid units which can be expanded in vitro, and upon differentiation, demonstrate the ability to give rise to regionally specialised intestinal epithelial stem cells. Intestinal epithelial stem cells can be obtained from intestinal biopsies or resection and, once isolated, can be maintained in vitro under defined culture conditions where they undergo functional differentiation and derived crypt-villus organoid structures [22, 23]. Moreover, intestinal epithelial cells can also be derived from induced pluripotent stem cells (iPSC) [24].

A different situation is currently found for smooth muscle cells. Although smooth muscle cells can be derived from biopsies taken from adult intestine, they generally fail to be expanded over sequential passages and ultimately undergo senescence. It is, therefore, relevant to explore the possibility of deriving “stem/progenitor” cells of intestinal smooth muscle, a critical cellular component of the neuromuscular apparatus, which will ultimately be required for functional restoration of contractility in any tissue engineered intestinal segment.

Recently, mesoangioblasts (MABs), which exist as multipotent stem/progenitor cells related to pericytes have been suggested as a powerful cell source for tissue regeneration [13–17]. When derived from adult tissue, MABs have been shown to generate both vascular and other mesodermal derivatives [18] including contributing to the regeneration of the neuromuscular wall of a bioengineered oesophagus [10]. Given this regenerative potential it is possible that MABs may provide an ideal cell source for regeneration of intestinal smooth muscle cells. Here, we show that MABs can be harvested from adult murine gut and expanded significantly maintaining their highly proliferative nature in culture. Both of these characteristics offer significant benefits as (1) isolating MABs from intestinal segments provides an easily accessible source of multipotent stem cells in children with IF and (2) the high proliferation rates observed in MABs isolated from intestinal segments may provide a mechanism to scale up to recreate large segments of intestinal tissue.

Previous studies of MABs as pericyte derivatives have proved difficult as there remain no unequivocal markers that allow for their identification. It is now assumed that pericytes can be identified by expression of at least two of the pericytes markers including platelet-derived growth factor receptor β, neuron-glial antigen 2, alanyl aminopeptidase, alpha-smooth muscle actin and desmin [17]. Here we demonstrate that murine MABs isolated from intestinal tissue express three known pericytes markers: neuron-glial antigen 2, alkaline phosphatase and platelet-derived growth factor receptor β. Hence, to the best of our knowledge, this is the first study reporting the isolation of MABs from the neuromuscular wall of the small intestine. Further studies are needed to establish whether those progenitors could also be isolated from human specimens.

Thus, this proof-of-principle study lays the foundation for the further characterisation of MABs as a possible cell source for intestinal smooth muscle regeneration. The ability of deriving smooth muscle progenitors from the intestinal wall may help establishing gut tissue engineering as a viable treatment of conditions such as IF.

## References

[1] Sanders KM, Koh SD, Ro S, Ward SM (2012). Regulation of gastrointestinal motility—insights from smooth muscle biology. Nat Rev Gastroenterol Hepatol.

[2] Kappus M, Diamond S, Hurt RT, Martindale R (2016). Intestinal failure: new definition and clinical implications. Curr Gastroenterol Rep.

[3] Duggan CP, Jaksic T (2017). Pediatric intestinal failure. N Engl J Med.

[4] Squires RH, Duggan C, Teitelbaum DH, Wales PW, Balint J, Venick R, Rhee S, Sudan D, Mercer D, Martinez JA, Carter BA, Soden J, Horslen S, Rudolph JA, Kocoshis S, Superina R, Lawlor S, Haller T, Kurs-Lasky M, Belle SH (2012). Natural history of pediatric intestinal failure: initial report from the Pediatric Intestinal Failure Consortium. J Pediatr.

[5] Boluda ER (2015). Pediatric small bowel transplantation. Curr Opin Organ Transplant.

[6] Grant CN, Mojica SG, Sala FG, Hill JR, Levin DE, Speer AL, Barthel ER, Shimada H, Zachos NC, Grikscheit TC (2015). Human and mouse tissue-engineered small intestine both demonstrate digestive and absorptive function. Am j physiol Gastrointest Liver Physiol.

[7] Urciuolo A, Urbani L, Perin S, Maghsoudlou P, Scottoni F, Gjinovci A, Collins-Hooper H, Loukogeorgakis S, Tyraskis A, Torelli S, Germinario E, Fallas MEA, Julia-Vilella C, Eaton S, Blaauw B, Patel K, De Coppi P (2018). Decellularised skeletal muscles allow functional muscle regeneration by promoting host cell migration. Sci Rep.

[8] Kitano K, Schwartz DM, Zhou H, Gilpin SE, Wojtkiewicz GR, Ren X, Sommer CA, Capilla AV, Mathisen DJ, Goldstein AM, Mostoslavsky G, Ott HC (2017). Bioengineering of functional human induced pluripotent stem cell-derived intestinal grafts. Nat Commun.

[9] Totonelli G, Maghsoudlou P, Garriboli M, Riegler J, Orlando G, Burns AJ, Sebire NJ, Smith VV, Fishman JM, Ghionzoli M, Turmaine M, Birchall MA, Atala A, Soker S, Lythgoe MF, Seifalian A, Pierro A, Eaton S, De Coppi P (2012). A rat decellularized small bowel scaffold that preserves villus-crypt architecture for intestinal regeneration. Biomaterials.

[10] Urbani L, Camilli C, Phylactopoulos DE, Crowley C, Natarajan D, McCann C, Pellegata AF, Urciuolo A, Aruta S, Signorelli M, Kiely D, Hannon E, Cossu G, Bonfanti P, De Coppi P (2018). Multi-stage bioengineering of a layered oesophagus with in vitro expanded muscle and epithelial adult progenitors. Nat Commun.

[11] Workman MJ, Mahe MM, Trisno S, Poling HM, Watson CL, Sundaram N, Chang CF, Schiesser J, Aubert P, Stanley EG, Elefanty AG, Miyaoka Y, Mandegar MA, Conklin BR, Neunlist M, Brugmann SA, Helmrath MA, Wells JM (2017). Engineered human pluripotent-stem-cell-derived intestinal tissues with a functional enteric nervous system. Nat Med.

[12] Schlieve CR, Fowler KL, Thornton M, Huang S, Hajjali I, Hou X, Grubbs B, Spence JR, Grikscheit TC (2017). Neural crest cell implantation restores enteric nervous system function and alters the gastrointestinal transcriptome in human tissue-engineered small intestine. Stem Cell Rep.

[13] Pellegata AF, Tedeschi AM, De Coppi P (2018). Whole organ tissue vascularization: engineering the tree to develop the fruits. Front Bioeng Biotechnol.

[14] Olson LE, Soriano P (2011). PDGFRbeta signaling regulates mural cell plasticity and inhibits fat development. Dev Cell.

[15] Collett GD, Canfield AE (2005). Angiogenesis and pericytes in the initiation of ectopic calcification. Circ Res.

[16] Dellavalle A, Sampaolesi M, Tonlorenzi R, Tagliafico E, Sacchetti B, Perani L, Innocenzi A, Galvez BG, Messina G, Morosetti R, Li S, Belicchi M, Peretti G, Chamberlain JS, Wright WE, Torrente Y, Ferrari S, Bianco P, Cossu G (2007). Pericytes of human skeletal muscle are myogenic precursors distinct from satellite cells. Nat Cell Biol.

[17] Armulik A, Genove G, Betsholtz C (2011). Pericytes: developmental, physiological, and pathological perspectives, problems, and promises. Dev Cell.

[18] Minasi MG, Riminucci M, De Angelis L, Borello U, Berarducci B, Innocenzi A, Caprioli A, Sirabella D, Baiocchi M, De Maria R, Boratto R, Jaffredo T, Broccoli V, Bianco P, Cossu G (2002). The meso-angioblast: a multipotent, self-renewing cell that originates from the dorsal aorta and differentiates into most mesodermal tissues. Development (Cambridge England).

[19] Tonlorenzi R, Dellavalle A, Schnapp E, Cossu G, Sampaolesi M (2007). Isolation and characterization of mesoangioblasts from mouse, dog, and human tissues. Curr Protoc Stem Cell Biol.

[20] Schneider CA, Rasband WS, Eliceiri KW (2012). NIH Image to ImageJ: 25 years of image analysis. Nat Methods.

[21] Elliott MJ, De Coppi P, Speggiorin S, Roebuck D, Butler CR, Samuel E, Crowley C, McLaren C, Fierens A, Vondrys D, Cochrane L, Jephson C, Janes S, Beaumont NJ, Cogan T, Bader A, Seifalian AM, Hsuan JJ, Lowdell MW, Birchall MA (2012). Stem-cell-based, tissue engineered tracheal replacement in a child: a 2-year follow-up study. Lancet.

[22] Sato T, Clevers H (2013). Growing self-organizing mini-guts from a single intestinal stem cell: mechanism and applications. Science.

[23] Sato T, Clevers H (2013). Primary mouse small intestinal epithelial cell cultures. Methods Mol Biol (Clifton NJ).

[24] Takahashi Y, Sato S, Kurashima Y, Yamamoto T, Kurokawa S, Yuki Y, Takemura N, Uematsu S, Lai CY, Otsu M, Matsuno H, Osawa H, Mizushima T, Nishimura J, Hayashi M, Yamaguchi T, Kiyono H (2018). A refined culture system for human induced pluripotent stem cell-derived intestinal epithelial organoids. Stem Cell Rep.

